# On-Device Execution of Deep Learning Models on HoloLens2 for Real-Time Augmented Reality Medical Applications

**DOI:** 10.3390/s23218698

**Published:** 2023-10-25

**Authors:** Silvia Zaccardi, Taylor Frantz, David Beckwée, Eva Swinnen, Bart Jansen

**Affiliations:** 1 Department of Electronics and Informatics (ETRO), Vrije Universiteit Brussel, 1050 Brussel, Belgium; taylor.frantz@vub.be (T.F.); bart.jansen@vub.be (B.J.); 2 Rehabilitation Research Group (RERE), Vrije Universiteit Brussel, 1090 Brussel, Belgium; david.beckwee@vub.be (D.B.); eva.swinnen@vub.be (E.S.); 3 IMEC, 3001 Leuven, Belgium

**Keywords:** HoloLens2, deep learning, machine learning, augmented reality, mixed reality, Windows Machine Learning, Unity Barracuda

## Abstract

The integration of Deep Learning (DL) models with the HoloLens2 Augmented Reality (AR) headset has enormous potential for real-time AR medical applications. Currently, most applications execute the models on an external server that communicates with the headset via Wi-Fi. This client-server architecture introduces undesirable delays and lacks reliability for real-time applications. However, due to HoloLens2’s limited computation capabilities, running the DL model directly on the device and achieving real-time performances is not trivial. Therefore, this study has two primary objectives: (i) to systematically evaluate two popular frameworks to execute DL models on HoloLens2—Unity Barracuda and Windows Machine Learning (WinML)—using the inference time as the primary evaluation metric; (ii) to provide benchmark values for state-of-the-art DL models that can be integrated in different medical applications (e.g., Yolo and Unet models). In this study, we executed DL models with various complexities and analyzed inference times ranging from a few milliseconds to seconds. Our results show that Unity Barracuda is significantly faster than WinML (*p*-value < 0.005). With our findings, we sought to provide practical guidance and reference values for future studies aiming to develop single, portable AR systems for real-time medical assistance.

## 1. Introduction

The integration of Artificial Intelligence (AI) in Augmented Reality (AR) systems is beneficial for a wide range of industrial and clinical applications [1]. AR systems often provide clinically relevant information to users about their surroundings, with information being derived from onboard image-based sensors. Most computer vision tasks for AR applications (e.g., image classification, object detection and pose estimation) benefit greatly from state-of-the-art Deep Learning (DL) models, specifically Convolutional Neural Networks (CNNs), enhancing performance and user experience [2]. Deep learning techniques commonly address tasks such as: object detection, frequently performed with real-time CNN-based DL models such as Yolo [3]; 3D semantic segmentation, which enables enhanced spatial understanding of indoor environments using point cloud data [4,5]; and hand gesture recognition [6,7], with promising implications in the field of touchless medical equipment [8]. However, integrating complex DL models into AR systems with limited computational capabilities can be challenging, particularly when real-time performance is needed [9].

AR systems in the medical field, e.g., for surgical assistance, ideally encompass several features [10]: (i) portability to accommodate various surgical and clinical settings, (ii) user-friendliness, (iii) real-time performance to provide timely and relevant information, and (iv) when feasible, voice-controlled functionality to leave the hands of the clinician free. AR Head-Mounted Displays (HMDs), or AR glasses, are currently being explored in the medical field as they can meet all the above-mentioned requirements [11,12,13]. Current studies focus on 3D visualization of pre/intra-operative imaging, such as blood vessels and brain MRI, to enhance decision-making processes and provide real-time feedback during complex surgical procedures (e.g., laparoscopy [14,15,16] and endoscopy [17,18]). Beyond the operating room, AR HMDs also show promise in medical training and education, superimposing virtual anatomical models and interactive simulations for an immersive learning experience [19,20,21,22]. Additionally, these devices are becoming pivotal in telemedicine, enhancing remote patient consultations with enriched visual aids and data overlays [23].

Among the numerous AR—or Mixed Reality (MR)—HMDs available on the market, HoloLens2 is well suited to the development of DL in AR applications due to its onboard processing capabilities. Its integrated “Holographic Processing Unit” (HPU) and suite of sensors enable complex functionalities, e.g., spatial mapping, hand and eye tracking, and voice control [24]. In the forthcoming years, cheaper, power-optimized, and more efficient processors are expected to become available on the market, further supporting the potential for combining AI and AR in standalone wearable AR devices [9]. A striking example of the rapid advancement in AR technology is the upcoming mixed-reality headset of Apple, Apple’s Vision Pro [25], which is believed to revolutionize the AR HMD user experience.

Despite HoloLens2 having an integrated GPU, its computing capabilities remain limited. Thus far, DL integration has mostly been performed by executing the DL models on an external server, which receives the input data (RGB or depth images) and sends the result back through a wireless connection. However, this architecture can be suboptimal for real-time medical applications due to the latency, instability, and accessibility issues associated with Wi-Fi connectivity. In particular, the reliance on external servers can be problematic in off-grid areas or during emergency situations. Another drawback of wireless connection is that it introduces additional radio frequency transmissions and requires supplementary computing infrastructure. This can be problematic in environments that are either sensitive or already limited in space, such as surgical rooms [26]. Therefore, in medical contexts, a straightforward, single-device AR system that ensures real-time performance can simplify the user experience and reduce potential technological pitfalls. In light of these limitations, this study aims to:

- Provide an overview of applications, identified in the literature, in which deep learning models are directly executed on the HoloLens2.
- Assess the feasibility of executing models with various complexity on HoloLens2.
- Compare two popular frameworks for neural network inference, Windows Machine Learning (WinML) and Unity Barracuda, in terms of inference time (i.e., the time that the model takes to make a prediction on a new image).
- Provide benchmark inference time values for state-of-the-art DL models for different medical applications (e.g., Yolo and Unet models for surgical guidance).

Our research builds upon the work of Lazar [27], which concluded that Barracuda was faster than WinML for a specific application using the Lenet5 model. In this study, we conduct a systematic evaluation of a broader range of DL models, providing reference values and relevant tests to quantify the performance of real-time medical applications on HoloLens2. With our findings, we aim to provide valuable technical guidance on how to integrate Machine Learning (ML) and DL models on HoloLens2.

## 2. Related Work

A review of academic works where ML/DL models are directly executed on HoloLens2 was performed. The review process involved a structured search on Web of Science, cross-referencing, and a hand search. For the structured search on Web of Science, a search string was used: “HoloLens” AND (“neural network” OR “deep learning” OR “machine learning” OR “artificial intelligence” OR “AI” OR “A.I.”). This yielded a total of 79 articles. After removing one duplicate, the following exclusion criteria were applied:

- Review papers (five papers excluded).
- Papers presenting datasets (1 paper excluded).
- Papers focusing on ML/DL model optimization (three papers excluded).
- Papers in which HoloLens2 is not used (three papers excluded).
- Papers in which the developed AR application does not integrate ML/DL models (25 papers excluded).
- Papers in which the integration of ML/DL models is performed by adopting a client-server architecture (39 papers excluded).

The filtering process resulted in only two papers in which ML/DL models are directly executed on HoloLens2 [28,29]. It also revealed that the majority of the works that integrate AI in HoloLens applications adopt a client-server architecture (e.g., Refs. [30,31]). Through cross-referencing and a hand search, we found six additional relevant ML/DL papers for HoloLens2 [27,32,33,34,35,36].

Table 1 summarizes the studies found in the literature, reporting their application context, the ML/DL model used and its task, the adopted framework (or Application Programming Interfaces, API), and the speed of the model (i.e., the inference time). Except for the study conducted by Lazar [27], all papers listed in Table 1 have opted for WinML as framework to integrate ML/DL models into HoloLens2. To the best of our knowledge, Lazar [27] is the only work in the literature where different approaches to run ML/DL models in HoloLens2 are implemented and compared. Table 1 also shows that the majority of the models are used to perform object detection [28,29,32,33,34,35,36]. These findings are in line with the literature review performed by Bohné [37], which focuses on studies that propose systems integrating machine learning and augmented reality. It is also worth mentioning that Bohné [37] suggests Unity Barracuda as framework to integrate ML/DL models in AR applications, being easy to implement and test. Von Atzigen [34], Doughty [29], and Doughty [28] applied object detection in specialized tasks within the medical field. Advanced techniques have been employed for the detection and pose estimation of surgical instruments [29], as well as for the prediction of surgery phases [28]. These specific use cases highlight the potential of deep learning models in real-time AR medical applications.

## 3. Materials and Methods

### 3.1. Microsoft HoloLens2

HoloLens2 is the second generation of Microsoft mixed reality headsets. It offers substantial improvements compared to its predecessor, HoloLens1: a custom-built Holographic Processing Unit (HPU), new sensors (an RGB camera, a depth camera, 4 visible light cameras, and an Inertial Measurement Unit (IMU)), and additional functionalities such as hand gestures and eye tracking. The user experience is further enhanced with a larger field of view (52°), improved battery life (3 h), and reduced weight (566 g) [38,39].

The HPU is a custom-designed ARM-based co-processor, developed to handle tasks related to data streams coming from all of HoloLens’ sensors. It is designed to offload work from the main processor, providing more efficient processing for the complex workloads involved in rendering holograms. The main processor, a Qualcomm Snapdragon 850 Compute Platform, has an ARM64 architecture and includes both a CPU and a GPU. The CPU is responsible for executing most of the computing instructions, while the GPU takes care of tasks related to rendering graphics [24]. An additional feature provided for users and developers is that GPU or CPU usage can be monitored using the Device Portal on the Windows platform, thus making it easier to manage and optimize application performance [40].

Along with the hardware improvements, HoloLens2 introduces a new version of the research mode, a C++ API that allows access to the raw streams of the sensors [41]. The research mode, coupled with the availability of benchmark data [42], further supports the use of HoloLens2 for real-time medical applications. Hololens2 spatial mapping (i.e., the ability to scan the surrounding environment in real-time and localize its position within it) and depth sensing have been extensively validated [43]. Moreover, the head-tracking capability has proven to provide accurate movement parameters for clinical gait analysis [44]. The processing capabilities and available tools make it possible to integrate deep learning models into real-time AR medical applications.

### 3.2. Deep Learning Integration on HoloLens2

Lazar [27] compares the performances of several frameworks (Windows Machine Learning (WinML), TensorFlow.NET, TensorFlow.js, and Barracuda) to integrate ML/DL models in HoloLens2. The study’s findings suggest that Barracuda is the optimal choice due to its faster inference and ease of implementation. In this study, we systematically assess the inference times of both WinML and Barracuda for a broader range of models. Our aim is not only to compare the two frameworks but also to examine the relationship between their performances and the complexities of different models.

**Figure 1:**
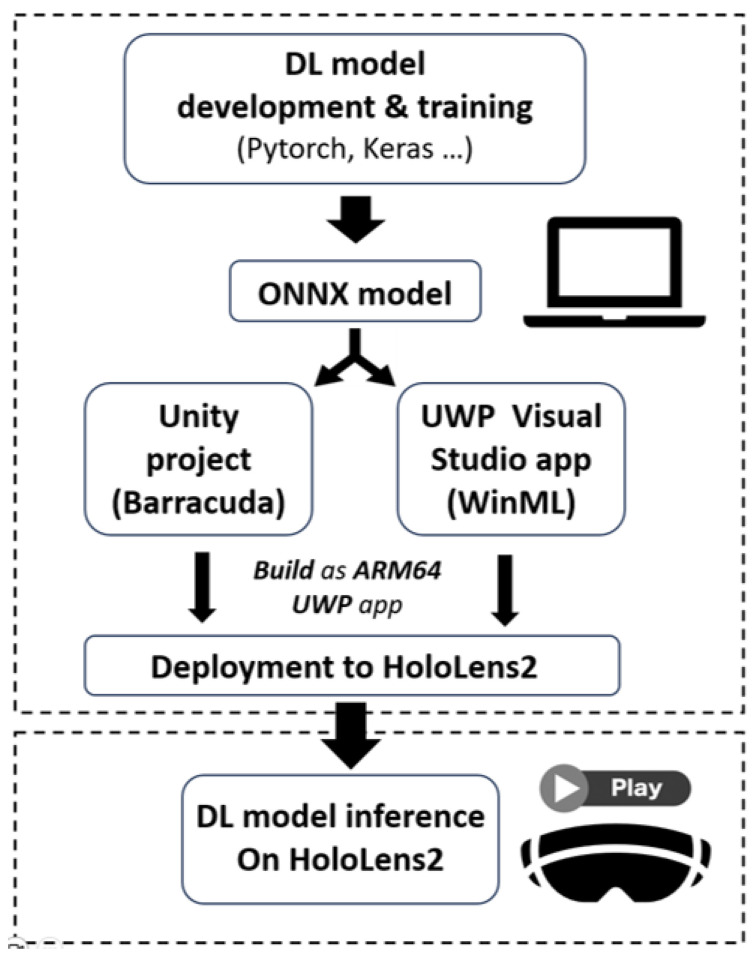
Overview of the pipeline for integrating DL models on HoloLens2.

To conduct our analysis, we developed two applications, one using WinML and one using Unity Barracuda. Both execute DL models in Open Neural Network Exchange Model (ONNX) format. Subsequently, the applications are deployed on HoloLens2, where the inference times are acquired for later processing. The pipeline for integrating DL models on HoloLens2 is depicted in Figure 1.

#### 3.2.1. Open Neural Network Exchange Model

The Open Neural Network Exchange (ONNX) is an open-source standard for representing machine learning models. It was introduced by Microsoft and Facebook with the goal of promoting interoperability within the machine-learning ecosystem [45]. This flexibility is achieved by defining a set of standardized operators—individual computations that make up the layers of a neural network—and opsets, which represent specific versions of these operators [46]. This standardization enables the same ONNX model to be run across different hardware and software environments.

In fact, a ML/DL model trained with one machine learning framework may not be compatible with another one (or may produce different results). By exporting the pre-trained model to ONNX, the model can be used in different projects by using the right execution tools. In this study, WinML and Barracuda represent the tools to execute pre-trained ONNX models on HoloLens2.

Machine learning frameworks such as TensorFlow, PyTorch, and Keras have native support for exporting to ONNX, and allow flexibility in the ONNX versioning. However, the choice of the ONNX opset version strictly depends on the specific Windows build targeted [47]. Moreover, not all versions of ONNX opset are compatible with all execution tools (i.e., WinML and Barracuda) and some operators are not supported at all.

#### 3.2.2. Unity Barracuda

Barracuda is a powerful machine learning inference library developed by Unity Technologies, designed for running DL models directly within Unity applications. Barracuda functionalities can be used in Unity applications by simply downloading the package from the Unity Package Manager [48]. To deploy the Unity application on HoloLens2, it must first be built as ARM64 Universal Windows Platform (UWP) app. Then, as every UWP application, it can be deployed using Visual Studio [49].

Although Barracuda is highly flexible and versatile, it currently does not support all model architectures and ONNX operations. However, the library effectively supports MobileNet v1/v2, Tiny YOLO v2, and U-Net models [50], which provide robust capabilities for a broad range of applications.

Barracuda can operate on a variety of device types, including CPU and GPU. In this study, we employed the worker type ComputedPrecompiled to execute the DL model on the HoloLens2 GPU. This worker precomputes certain tasks, which optimizes the model’s performance and allows for efficient utilization of the GPU resources [51].

#### 3.2.3. Windows Machine Learning

Windows Machine Learning (WinML) is a Microsoft API that enables developers to run ML/DL models natively on Windows devices, including the HoloLens2 [52]. It comes with the standard Windows 10 SDK, which can be installed in Visual Studio through the Visual Studio Installer. In this study, the API was used in C# UWP applications, which, once built for ARM64 platform, were deployed on HoloLens2 [53]. WinML is well-known within a broad user community, supports many DL architectures and offers comprehensive documentation.

Similarly to Barracuda, WinML can utilize different hardware resources for model execution (CPU and GPU), as the API allows the selection of the device to evaluate the model on. For this study, the DirectXHighPerformance device was selected for execution. DirectXHighPerformance refers to the most powerful GPU available on the system. This choice allowed us to maximize the high-performance HoloLens2 capabilities for inference computations [54].

In this study, WinML was used in UWP applications, due to the straightforward implementation. However, given Unity’s capability to support a multitude of platforms, it is worth mentioning that the use of WinML in Unity applications is possible. Such integration requires the use of Dynamic Link Libraries (DLLs), potentially decreasing the ease of implementation and debugging.

#### 3.2.4. Evaluation Metric: Inference Time

We chose the inference time as the metric to evaluate the performances of WinML and Barracuda. Inference time refers to the duration between executing a model on a single image, measured from the start to the end of the execution. In our experiments, we simulate the typical use case scenario in which the model integrated into HoloLens2 processes real-time images captured by the HoloLens2 colored camera, one image at a time.

To measure the inference time, we utilize the C# Stopwatch class [55]. The stopwatch is started immediately before the model execution and stopped right after its completion. For WinML, the execution code is as follows:

output = await model. EvaluateAsync(image);

This code invokes the EvaluateAsync method, which internally calls the CreateFromStreamAsync method, where the actual inference is performed [54].

For Barracuda, the inference time is calculated as the duration of executing the following lines of code:

output = engine. worker. Execute(image). PeekOutput();
engine. worker. FlushSchedule(true);

The FlushSchedule method with flag set to True is needed in order to block the main thread until the execution is complete [51].

By measuring the inference time using these methods, we can accurately assess the performance of WinML and Barracuda in terms of execution speed.

### 3.3. Experimental Design

The experimental study consists of two phases. In the first phase, we systematically assess the performance of WinML and Barracuda in terms of inference time for CNN models with increasing complexities. In the second phase, we evaluate the inference times of both frameworks for State Of The Art (SOTA) DL models.

To measure the mean inference time for all models, we employ two template applications, one for each framework. These applications read 200 images stored in a local folder and perform model inference on each image using a for loop. After completing the loop, the inference times are recorded in a .txt file. Each experiment is repeated 5 times, resulting in 1000 samples of inference times for each framework and model.

#### 3.3.1. Impact of Model Complexity on Inference Time

To investigate the performances of WinML and Barracuda, we created multiple DL models with varying complexities. The architecture of the models is composed of stacked convolutional layers; the input layer size is 256 × 256 × 3, and the consequent convolutional layers have a kernel size of 3 × 3. By adjusting the number of layers, i.e., the depth of the model, and the number of filters, we were able to create models with different architectures. Each model was then exported to ONNX format. To evaluate the computational complexity of the models, we determined the number of Multiply-Accumulates (MACs) and the number of parameters. We created two distinct groups of CNN models:

- Group A. The models belonging to group A have similar complexity in terms of MACs and parameters, but different depths and number of filters (see Table 2).
- Group B. The models belonging to group B have the same depth, but increasing MACs and number of parameters (see Table 3).

By following this methodology, we aimed to provide a comprehensive analysis of DL model variations in terms of size, computational complexity (MACs and parameters), and architecture (depth and number of filters).

#### 3.3.2. Feasibility of SOTA Models Integration

The second phase of the experimental study aims to assess the performance of WinML and Barracuda in executing state-of-the-art (SOTA) models on the HoloLens2 device. To conduct this evaluation, we selected a subset of the models listed in Table 1:

- Lenet-5, from the work of Lazar [27]. The model was implemented using a public GitHub repository [56] and trained on the MNIST dataset, a large database of handwritten digits [57]. The model is trained to recognize digits between 0 and 9 from grayscale images of 32 × 32 pixels.
- SurgeonAssist-Net [28]. The model infers the poses of a drill and of the surgeon’s hand from RGB images of 224 × 224 pixels. The ONNX model, pre-trained on the Colibrì dataset [58], is available in the official GitHub repository of the paper [28]. The model version “PyTorch_1.4” was used in this study.
- HMD-EgoPose [29]. The model predicts the surgical phase from RGB images of 256 × 256 pixels. The ONNX model, pre-trained on the Cholec-80 dataset [59], is available in the official GitHub repository of the paper [29].
- Yolov4-Tiny [34,36]. The model performs object detection in RGB images of 416 × 416 pixels. For our study, we utilized a pre-trained version of the model on the Pascal Visual Object Classes (VOC) dataset [60], which is available in a public GitHub repository [61]. The model was trained to detect 20 different classes.

In addition, we assessed the inference time of two models that were not found in our literature review:

- Resnet50 model [62] for 2D Human Pose Estimation (HPE). The model estimates the 2D poses of multiple people from RGB images with variable sizes (in this study, the input of the model are images of 256 × 256 pixels). Mills [63] provides a pre-trained ONNX model in a public GitHub repository.
- Unet model. The pre-trained ONNX model was obtained from a public GitHub repository [64]. The model performs semantic segmentation of RGB images (256 × 256 pixels). The model version “u2netp.onnx” was used in this study.

We maintained consistency by utilizing the same template applications and methodology (5 repetitions of 200 images for each model) as in the first experimental phase. In order to minimize additional factors that could contribute to the inference time and application FPS (Frames Per Second), we minimized the image processing and post-processing steps, and rendering was intentionally avoided.

#### 3.3.3. Software and Library Versions

The HoloLens2 device used in our experiments was running on OS build 20348.1543. The C# UWP apps that utilize WinML were developed using Visual Studio Community 2019, with Windows 10 build 19041 (Version 2004). WinML is part of the Windows 10 SDK (MSVC v142). The Unity projects that employ Barracuda have an editor version of 2021.3, and use Barracuda version 3.0. The CNNs models were created using a custom-made Python script (Python version 3.9, TensorFlow library version 2.12, ONNX version 1.14). The library “onnx_tools” (version 0.3) was used for ONNX model profiling. All ONNX models have opset version 10, except for Lenet-5, SurgeonAssist-Net and Unet, which have opset version 9, and Yolov4-Tiny model, which has opset version 8.

## 4. Results

This section presents the outcomes of the experiments outlined in Section 3.

### 4.1. Impact of Model Complexity on Inference Time: Results

**Figure 2:**
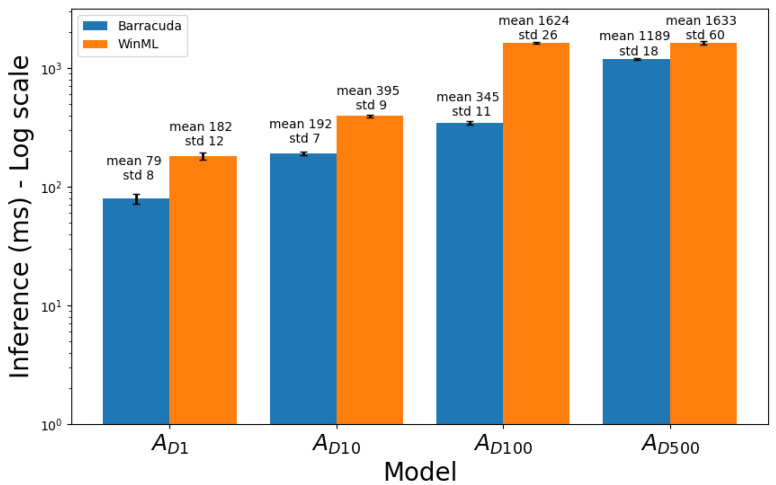
Comparison of mean inference times for models of Group A. The bars represent the average inference time across five repetitions of 200 images with each model. The values on top of each bar indicate the mean inference time and the average standard deviation (in milliseconds) across the five repetitions. The y-axis is in logarithmic scale.

**Figure 3:**
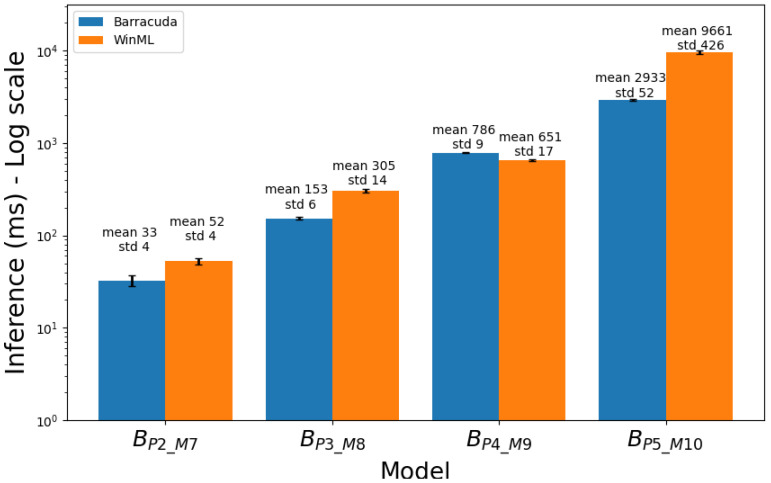
Comparison of mean inference times for models of Group B. The bars represent the average inference time across 5 repetitions of 200 images with each model. The values on top of each bar indicate the mean inference time and the average standard deviation (in milliseconds) across the five repetitions. The y-axis is in logarithmic scale.

Table 4 and Table 5 present the average inference times for models in Group A and Group B, respectively. The tables provide a comparison of each model’s performance, with inference times and standard deviations indicated for both WinML and Barracuda. Figure 2 and Figure 3 present the corresponding bar diagrams.

The Pearson correlation test was applied to evaluate the linear relationship between model depth and inference time across the Group A models. For WinML, the Pearson correlation coefficient was 0.72, with a statistically significant *p*-value (<0.005). For Barracuda, the Pearson correlation coefficient was 0.99, with a statistically significant *p*-value (<0.005).

The Pearson correlation test was applied to evaluate the linear relationship between model MACs and number of parameters, separately, and inference time across the Group B models. For both WinML and Barracuda, the Pearson correlation coefficient between MACs and inference time, as well as the correlation coefficient between the number of parameters and inference time, were 0.99, with statistically significant *p*-values (<0.005).

### 4.2. Windows Machine Learning vs. Unity Barracuda

In this analysis, we compare the inference times of WinML and Barracuda for all the models (Group A and Group B). For each model, we considered the five repetitions of the two frameworks as two independent samples. Due to the non-normality of the data (the normality check was performed with the function “normaltest” of scipy Python library [65], which is based on D’Agostino and Pearson’s [66]) the non-parametric Mann–Whitney U test was applied. In all cases, the test returned a *p*-value less than 0.005, revealing a statistically significant difference between the inference times of WinML and Barracuda across all models.

Following the statistical test, we computed the mean ratio of Barracuda’s inference time to that of WinML for each model. Barracuda outperformed WinML for the majority of the models. The mean ratio was less than 1 for 7 out of 8 models, implying a faster inference time for Barracuda. Conversely, only one model ($B_{P4\_M9}$) exhibited a mean ratio exceeding 1, implying a faster inference time for WinML in that instance.

### 4.3. Feasibility of SOTA Models Integration: Results

Table 6 reports the mean inference times recorded when executing the SOTA models with both WinML and Barracuda. However, it was not possible to test the SurgeonAssist-Net model with Barracuda, as the model includes Gated Recurrent Units (GRUs) which are not supported. Our results can be compared with the inference times reported by the original authors:

- SurgeonAssist-Net. Our results show higher inference times with WinML on GPU than the original authors reported for WinML on CPU, which they reported as 219 ms in the paper and estimated between 200 and 350 ms in their GitHub repository.
- HMD-EgoPose. We recorded inference times of around 1 s when using WinML on GPU, similar to the inference times reported by the author in CPU. However, when executed with Barracuda, the recorded inference times were notably shorter at 384 ms.
- Yolo-v2Tiny. We recorded inference times of 1.3 s using WinML on GPU, comparable with the literature (the inference times reported by von Atzigen [34] and Hamilton [36] are, respectively, 900 ms and 1.7 s). Using Barracuda, the inference times decrease to 630 ms.

For Lenet-5, the authors reported inference times for a batch of 10,000 images, which is not directly comparable with our individual inference times. Regarding Resnet50, we found no reference values as, to the best of our knowledge, no previous works have executed a Resnet50 model directly on HoloLens2. Similarly, for the Unet model, we could not find any reference values. The works we reviewed—the research paper by Zakaria [35] and a GitHub repository [64]—did not provide such information.

## 5. Discussion

Our results indicate that both the complexity of deep learning models and the choice of framework significantly influence inference time. We investigated the impact of model complexity, and found a strong positive correlation between model depth, number of parameters, MACs, and inference time. These findings align with theoretical expectations and prior research: (more) complex models generally require more computational power and thus, more time to infer. We also found that Barracuda consistently outperformed WinML, except for one of the tested models ($B_{P4\_M9}$). This may be due to differences in the framework implementations that are beyond the scope of this study.

Table 6 shows striking examples of performance improvement when opting for Barracuda over WinML. In particular, the HMD-EgoPose model (originally deemed unsuitable for real-time applications due to an inference time of 1 s with WinML) showed an improved speed of 384 ms. During surgery, a lag or delay in recognizing the drill’s pose can interfere with the precision of the incision. With Barracuda, the inference speed for HMD-EgoPose nearly tripled compared to WinML, greatly enhancing its potential utility in surgical procedures. Another compelling example is the inference time achieved with Barracuda for the YoloV2-Tiny model. In our tests, Barracuda registered an inference time of 630 ms, which is less than half of WinML’s 1330 ms. Notably, the inference of Barracuda is considerably faster than that previously reported in the literature, with one study [34] reporting 1 s, and another [36] reporting 1.7 s. Von Atzigen [34] successfully employed YoloV2-Tiny for the detection of implanted pedicle screw heads during spinal fusion surgery. However, the authors acknowledge that the low application frame rate is a limitation of their study. As for HMD-EgoPose, adopting Barracuda could potentially help translate von Atzigen’s [34] proof-of-concept application in clinical practice.

Our results, in line with the works of Lazar [27] and Bohné [37], strongly suggest exploring Barracuda as an inference framework. While WinML may support a broader range of DL architectures, Barracuda allows for faster model inference and is easier to integrate in Unity applications—a valuable feature given Unity’s support for the development of Apple Vision Pro applications [67]. Our results suggest that, by adopting Barracuda to execute DL models directly on HoloLens2: (i) high application frame rates (>30 fps) can be achieved by models with MACs less than $10^{7}$, such as Lenet5; (ii) more complex models, such as EfficientNetB0, are likely to yield an application frame rate of only a few fps; (iii) models with a number of MACs of the order of $10^{10}$, such as Resnet50 and Unet, will likely exhibit inference times of the order of seconds.

### Limitations and Future Research

Despite our results demonstrating the feasibility of integrating SOTA models into HoloLens2, there are several study limitations. Firstly, it is limited to a specific set of DL models and conditions. Besides model complexity and framework choice, software versions can also greatly influence inference time. Table 6 reveals a discrepancy in the inference times of SurgeonAssist-Net using WinML between our study and that of the original authors. It is possible that the authors explored a range of builds and versions to fine-tune performance, an approach we did not adopt in our analysis. Secondly, while the inference time represents the execution speed of the models, the overall application frame rate can be influenced by other factors (e.g., rendering and image processing).

It is also important to acknowledge that, although the execution of SOTA models is faster with Barracuda, it is not yet adequate for all applications. A relevant example is HPE; performing real-time (>30 fps) HPE on HoloLens2 could enable physiotherapists to intuitively visualize the motion parameters of their patients, such as their range of motion, rather than relying solely on 2D screen displays. However, Resnet50 yielded an inference time of 700 ms, corresponding to 1.4 fps (Table 6). Moreover, executing SOTA models on-device may not be feasible for image-guided surgery AR applications requiring high frame rate and real-time feedback. However, the performances of SOTA models can still be adequate for surgical planning, needle insertion, and medical training.

Future studies should explore optimization techniques (e.g., post-training quantization [68]) for faster inference, and quantify their impact on model accuracy. In addition, newer DL architectures (e.g., Vision Transformers [69]) should be investigated. Executing DL models in Unity applications using Barracuda can ease the transition from HoloLens2 to future AR HMDs—as the upcoming Apple Vision Pro—broadening the horizon for real-time medical applications.

## 6. Conclusions

In conclusion, this study presents a systematic evaluation of the influence of model complexity for deep learning models running directly on HoloLens2. Additionally, we compared the performances of two popular inference frameworks—Windows Machine Learning and Unity Barracuda. Our results showed that model complexity in terms of depth, parameters, and MACs positively correlates with inference time. Furthermore, we found significant differences in the performance of WinML and Barracuda frameworks, with Barracuda generally yielding faster inference times. With our work, we sought to provide technical guidance and reference values for future HoloLens2 applications that aim to execute DL models directly on the device.

## Figures and Tables

**Table 1:** Overview of AR applications executing ML/DL models directly on Microsoft HoloLens2.

Paper	Application	Model	Model Task	API	Speed
Zakaria [35]	Infrastructure (Bridge inspection)	Yolov5 Unet	Object detection Semantic segmentation	-	-
Quin [32]	Manufacturing (Assembly)	Yolov5	Object detection	WinML	370 ms
Zao [33]	Manufacturing (Assembly)	Yolov4-Tiny	Object detection	-	360 ms
Hamilton [36]	Medical (Daily Reminder system)	Yolov2-Tiny	Object detection	WinML	1773 ms
von Atzigen [34]	Medical (Surgical navigation)	Yolov2-Tiny	Object detection	WinML	900 ms
Doughty [29]	Medical (Surgical navigation)	HMD-EgoPose (EfficientNetB0 backbone)	Pose estimation	WinML	≈1 s
Doughty [28]	Medical (Surgical navigation)	SurgeonAssist-Net (EfficientNet-Lite-B0 backbone)	Action recognition	WinML	219 ms
Lazar [27]	Benchmark (APIs performances)	LeNet5	Image classification	WinML TensorFlow.js Barracuda	− 3428 ms ${}^{1}$ − 1035 ms ${}^{1}$ − 183 ms ${}^{1}$

^1^ Inference time for a batch of 10,000 images.

**Table 2:** Specifications of Group A ONNX models, listed by their unique identifier (ID), model size in megabytes (Mb), total number of parameters (# params), Multiply-Accumulates (# MACs), number of filters (# filters), and depth of the network (depth). The ID of each model indicates its depth, with $A_{D1}$ representing a depth of 1, $A_{D10}$ a depth of 10, and so on.

ID	Model Size (Mb)	# Params	# MACs	# Filters	Depth
$A_{D1}$	0.1	3024	212,860,928	112	1
$A_{D10}$	0.2	5400	364,380,160	8	10
$A_{D100}$	2.1	3618	263,323,648	8	100
$A_{D500}$	1.7	4522	361,627,648	1	500

**Table 3:** Specifications of Group B ONNX models, listed by their unique identifier (ID), model size in megabytes (Mb), total number of parameters (# params), Multiply-Accumulates (# MACs), number of filters (# filters), and depth of the network (depth). The ID of each model indicates the logarithm (base 10) of the order of magnitude of its number of parameters and MACs (e.g., $B_{P2\_M7}$ represents a model with a number of parameters of the order of $10^{2}$, and a number of MACs of the order of $10^{7}$).

ID	Model Size (Mb)	# Params	# MACs	# Filters	Depth
$B_{P2\_M7}$	0.04	$10^{2}$	$10^{7}$	1	10
$B_{P3\_M8}$	0.1	$10^{3}$	$10^{8}$	6	10
$B_{P4\_M9}$	0.7	$10^{4}$	$10^{9}$	16	10
$B_{P5\_M10}$	1.3	$10^{5}$	$10^{10}$	64	10

**Table 4:** WinML and Barracuda inference times for Group A models. All models have similar values of MACs and number of parameters (see Table 2).

Model Specifications			Inference Time (ms)	
**ID**	**# Filters**	**Depth**	**WinML**	**Barracuda**
$A_{D1}$	112	1	182 ± 12	79 ± 8
$A_{D10}$	8	10	395 ± 9	192 ± 7
$A_{D100}$	8	100	1624 ± 26	345 ± 11
$A_{D500}$	1	500	1633 ± 60	1189 ± 18

**Table 5:** WinML and Barracuda inference times for Group B models. All models have a depth of 10.

Model Specifications				Inference Time (ms)	
**ID**	**# Params**	**# MACs**	**# Filters**	**WinML**	**Barracuda**
$B_{P2\_M7}$	$10^{2}$	$10^{7}$	1	52 ± 4	33 ± 4
$B_{P3\_M8}$	$10^{3}$	$10^{8}$	6	305 ± 14	153 ± 6
$B_{P4\_M9}$	$10^{4}$	$10^{9}$	16	651 ± 17	786 ± 9
$B_{P5\_M10}$	$10^{5}$	$10^{10}$	64	9661 ± 426	2933 ± 52

**Table 6:** WinML and Barracuda inference time with SOTA models. The “Lit. WinML” column presents the inference times as reported by the original authors.

Model Specifications					Inference Time (ms)		
**ID**	**Size (Mb)**	**# Params**	**# MACs**	**Depth**	**WinML**	**Barracuda**	**Lit. WinML**
Lenet-5 [27]	0.25	$10^{5}$	$10^{6}$	5	5 ± 2	5 ± 8	-
SurgeonAssist-Net [28]	15	$10^{6}$	$10^{8}$	50	465 ± 17	NA	219
EgoPose [29]	16	$10^{6}$	$10^{9}$	1194	1164 ± 43	384 ± 14	≈1000
Yolov2-Tiny [34,36]	62	$10^{7}$	$10^{9}$	25	1330 ± 56	630 ± 32	900 [34] 1773 ± 34 [36]
Resnet50 [63]	90	$10^{7}$	$10^{10}$	98	1842 ± 64	701 ± 14	-
Unet [64]	4	$10^{6}$	$10^{10}$	259	4707 ± 162	3023 ± 42	-

## Data Availability

The data presented in this study are available on request from the corresponding author.
